# Postconditioning with rosuvastatin reduces myocardial ischemia-reperfusion injury by inhibiting high mobility group box 1 protein expression

**DOI:** 10.3892/etm.2013.1362

**Published:** 2013-10-24

**Authors:** XIANJIN DU, XIAORONG HU, JIE WEI

**Affiliations:** 1 Department of Emergency, Renmin Hospital of Wuhan University, Wuhan, Hubei 430060, P.R. China; 2 Department of Cardiology, Renmin Hospital of Wuhan University, Wuhan, Hubei 430060, P.R. China; 3 Department of Cardiology, Huangshi Central Hospital, Affiliated Hospital of Hubei Polytechnic University, Huangshi, Hubei 435000, P.R. China

**Keywords:** rosuvastatin, myocardial ischemia, reperfusion, high mobility group box 1 protein

## Abstract

High mobility group box 1 protein (HMGB1) plays an important role in myocardial ischemia-reperfusion (I/R) injury. Rosuvastatin (RS) preconditioning has been reported to reduce myocardial I/R injury. The aim of this study was to investigate whether postconditioning with RS is able to reduce myocardial I/R injury by inhibiting HMGB1 expression in rats. Anesthetized male rats were subjected to ischemia for 30 min and treated once with RS (10 mg/kg, i.v.) 5 min prior to reperfusion for 4 h. Lactate dehydrogenase (LDH), creatine kinase (CK) and superoxide dismutase (SOD) activities, malondialdehyde (MDA) levels and infarct size were measured. HMGB1 expression was assessed by immunoblotting. The results showed that RS postconditioning significantly decreased the infarct size and the activities of LDH and CK following 4 h reperfusion (all P<0.05). RS postconditioning also significantly inhibited the increase of MDA levels and the reduction of SOD activity (both P<0.05). RS postconditioning was able to significantly inhibit the HMGB1 expression induced by I/R. The present study suggested that postconditioning with RS reduces myocardial I/R injury, which may be associated with the inhibition of HMGB1 expression.

## Introduction

High mobility group box 1 protein (HMGB1), a non-chromosomal nuclear protein, may be passively released by necrotic, apoptotic or positively activated innate immune cells (including macrophages and monocytes) (1,2). HMGB1 has been demonstrated to be a pro-inflammatory cytokine in certain cardiovascular diseases (3–6). A previous study showed that HMGB1 acts as an early mediator of inflammation during myocardial ischemia-reperfusion (I/R) as well as classical early pro-inflammatory cytokines, including tumor necrosis factor-α (TNF-α) and interleukin-6 (IL-6), and may promote the release of TNF-α and IL-6. By contrast, HMGB1 A box peptide (a specific HMGB1 antagonist) was able to attenuate myocardial I/R injury and inhibit the release of TNF-α and IL-6 (3). The inflammatory response is considered to be a critical factor of myocardial I/R injury (7,8). These results demonstrate that HMGB1 may play an important role in myocardial I/R injury.

Rosuvastatin (RS) is a drug that reduces cholesterol production by inhibiting 3-hydroxy-3-methylglutaryl coenzyme A (HMG-CoA) reductase. Previous studies have shown that preconditioning with RS provides cardioprotective effects, such as reducing myocardial I/R injury (9–13). However, whether postconditioning with RS is able to reduce myocardial I/R injury and its mechanism of action remains unclear. Previous studies have indicated that statins are able to inhibit HMGB1 expression (14,15). Therefore, the hypothesis that postconditioning with RS reduces myocardial I/R injury by inhibiting HMGB1 expression was tested in a rat myocardial I/R model in the present study.

## Materials and methods

### Animal preparation and experimental design

The experiment protocol conformed to the Guide for the Care and Use of Laboratory Animals published by the US National Institutes of Health (NIH Publication, revised 1996) and was approved by the Animal Care and Use Committee of Wuhan University, Wuhan, China. Male Sprague-Dawley rats (250–300 g) were randomly assigned into three groups receiving the following treatments: Group 1, sham-operated control (SO; n=10) in which the rats were subjected to surgical manipulation without the induction of myocardial ischemia; Group 2, ischemia and reperfusion (I/R; n=15) in which the rats were subjected to left anterior descending coronary artery (LAD) occlusion for 30 min followed by reperfusion for 4 h; Group 3, RS + I/R (RS - I/R; n=15) in which the rats were subjected to LAD occlusion for 30 min, followed by reperfusion for 4 h. Rats were treated once with RS (10 mg/kg, i.v.) (9,13) 5 min prior to reperfusion. RS was dissolved in sterile saline.

Following anesthesia with sodium pentobarbital (45 mg/kg, i.p.), the rats were ventilated artificially with a volume-controlled rodent respirator (TKR 400H; Jiangxi Teli Anesthesia Breathing Equipment Co., Ltd., Nanchang, China) at 70 strokes per minute. The rats were placed on an electric heating pad to maintain the body temperature at 37°C. Heparin (200 IU/kg, i.v.) was administered prior to ischemia. Lead-II of the electrocardiogram was monitored with subcutaneous stainless steel electrodes. The electrocardiogram was monitored using a computer-based EP system (LEAD2000B; Jinjiang Ltd., Chengdu, China).

A thoracotomy through a left parasternal incision was performed to expose the anterior wall of the left ventricle. A 4-0 silk suture on a small curved needle was passed through the myocardium beneath the middle segment of the LAD branch coursing down the middle of the anterior wall of the left ventricle. A small vinyl flake was passed into both ends of the suture, which was then fixed by clamping the tube with a mosquito hemostat. A successful myocardial I/R model was confirmed by ST segment elevation in Leads-II and regional cyanosis of the myocardial surface. The rats underwent a 30-min occlusion of the LAD, followed by a 4-h reperfusion.

### Assessment of infarct size

Infarct size was established by 2,3,5-triphenyltetrazolium chloride (TTC; Sigma-Aldrich, St. Louis, MO, USA) staining. Briefly, after reperfusion the LAD was occluded again and 2 ml 1.0% Evans blue dye was injected via the femoral vein. Each heart was then sliced horizontally to yield five slices. The slices were incubated in 1% TTC for 15 min at 37°C. The infarct area (white) and the area at risk (red and white) from each section were measured using an image analyzer (Image-Pro Plus 3.0; Media Cybernetics, Silver Spring, MD, USA). Infarct size was expressed as a percentage of the risk area volume (%, infarct size/risk area).

### Assessment of myocardial injury

To assess the lactate dehydrogenase (LDH) and creatine kinase (CK) activities, blood samples were collected and centrifuged at 1,000 × g for 20 min. Standard techniques using the LDH Assay kit and CK Assay kit according to the manufacturer’s instructions (Nanjing Jiancheng Bioengineering Institute, Nanjing, China) were used in the analyses. Values are expressed in international units (IU) per liter.

### Measurement of myocardial malondialdehyde (MDA) levels and superoxide dismutase (SOD) activity

The MDA concentration and SOD activity in myocardial tissue were measured using an MDA Assay kit and SOD Assay kit (Nanjing Jiancheng Bioengineering Institute) according to the manufacturer’s instructions. The former was used as an index of oxygen free radicals and the latter as an indicator of the lipid superoxide level in the myocardium.

### Immunoblotting analysis

Pulverized frozen ischemic areas of left ventricle samples were analyzed by quantitative immunoblotting using a HMGB1 antibody (Santa Cruz Biotechnology, Inc. Santa Cruz, CA, USA) as described in a previous study (16). The expression of protein was normalized to glyceraldehyde-3-phosphate dehydrogenase (GAPDH) expression.

### Statistical analysis

Statistical analysis was performed with SPSS 16.0 (SPSS, Inc., Chicago, IL, USA). All values were expressed as mean ± SD. One-way ANOVA or Welch ANOVA were used for comparisons among groups and the least-significant difference or Dunnett T3 test was used for post-hoc multiple comparisons. P<0.05 was considered to indicate a statistically significant result.

## Results

### Infarct size

**Figure 1:**
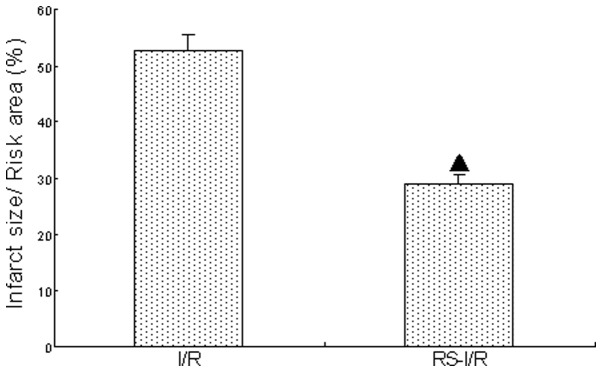
Effect of RS postconditioning on infarct size during I/R (n=5). ^▲^P<0.05 vs. the I/R group. RS, rosuvastatin; I/R, ischemia-reperfusion.

After 4 h of reperfusion, the infarct size induced by I/R was decreased by RS postconditioning compared with that in the I/R group (28.9±3.7 vs. 52.8±4.4%, P<0.05; Fig. 1).

### LDH and CK activities

**Figure 2:**
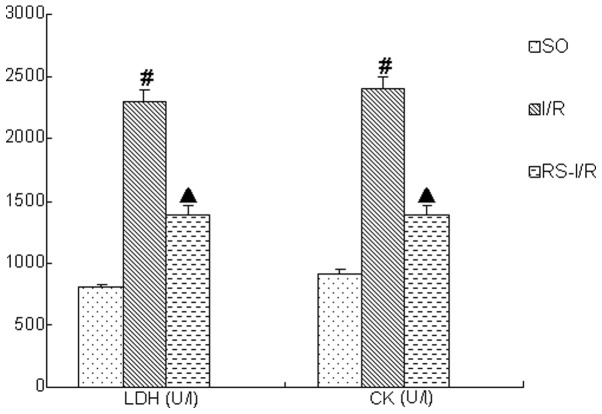
Effect of RS postconditioning on LDH and CK during I/R (n=10). ^#^P<0.05 vs. the SO group; ^▲^P<0.05 vs. the I/R group. LDH, lactate dehydrogenase; CK, creatine kinase; SO, sham-operated control; RS, rosuvastatin; I/R, ischemia-reperfusion.

After 4 h of reperfusion, the LDH and CK activities in the I/R group were significantly increased compared with those in the SO group (P<0.05). However, the increases of LDH and CK activities were significantly reduced by RS postconditioning (both P<0.05; Fig. 2).

### MDA levels and SOD activity

**Figure 3:**
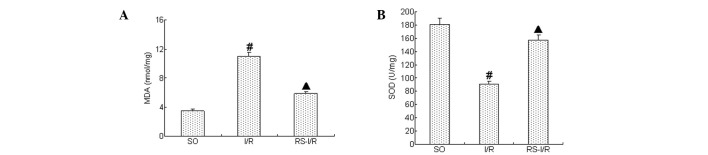
Effect of RS postconditioning on (A) MDA and (B) SOD during I/R (n=5). ^#^P<0.05 vs. SO group; ^▲^P<0.05 vs. I/R group. MDA, malondialdehyde; SOD, superoxide dismutase; SO, sham-operated control; RS, rosuvastatin; I/R, ischemia-reperfusion.

After 4 h of reperfusion, the MDA level in the I/R group was significantly increased while the SOD activity was significantly decreased compared with that in the SO group (P<0.05). The increase of the MDA level and the reduction of the SOD level were significantly inhibited by RS postconditioning (both P<0.05; Fig. 3).

### HMGB1 expression

**Figure 4:**
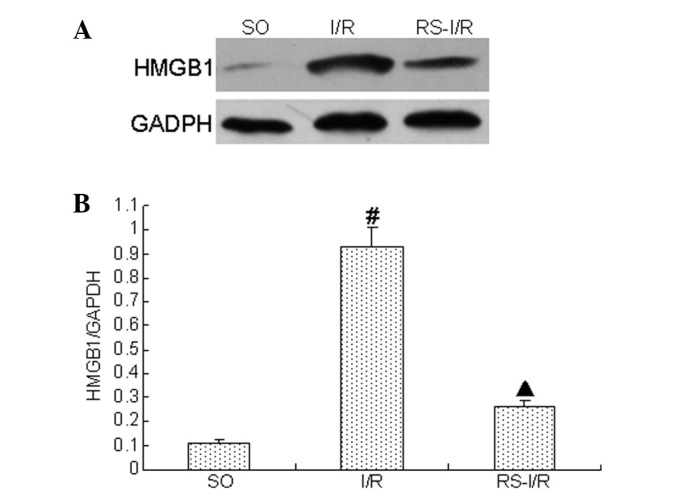
Effect of RS postconditioning on HMGB1 expression during I/R (n=5). (A) HMGB1 expression in ischemic areas of left ventricle samples were examined and analyzed by quantitative immunoblotting. The expression of protein was normalized to GAPDH expression. (B) Densitometric analysis of results in (A) following normalization to GAPDH. The results presented are the means ± SD of three independent experiments. ^#^P<0.05 vs. SO group; ^▲^P<0.05 vs. I/R group. HMGB1, high mobility group box 1 protein; SO, sham-operated control; RS, rosuvastatin; I/R, ischemia-reperfusion.

As shown in Fig. 4, HMGB1 expression was markedly increased after 4 h of reperfusion (P<0.05), and this increase in expression was significantly inhibited by RS postconditioning (P<0.05).

## Discussion

RS is relatively hydrophilic; in contrast to lipophilic statins, it has reduced access to numerous cell types throughout the body by diffusion across cell membranes (17). The uptake of RS is extremely high in hepatocytes and cells that express active transporters for anionic compounds, such as RS. This property, associated with the higher affinity of RS for the active site of HMG-CoA reductase than other statins (17). However, the cardioprotective effects of RS have clearly been documented, such as the reduction of myocardial I/R injury (9–13). Jones *et al*(9) observed that RS preconditioning may reduce myocardial I/R injury by mediating endothelial nitric oxide production. Recently, Ke *et al*(13) showed that RS preconditioning is able to reduce the accumulation of inflammatory cells and decrease infarct size. Bell *et al*(18) showed that atorvastatin postconditioning was able to protect against myocardial I/R injury independent of lipid lowering. In the present study, RS postconditioning reduced myocardial I/R injury and HMGB1 expression. The inflammatory response is considered to be a critical factor of myocardial I/R injury (7,8). Previous studies have demonstrated that HMGB1 is a critical pro-inflammatory cytokine for myocardial I/R injury (3,8). Therefore, we suggest that RS postconditioning reduces myocardial I/R injury by inhibiting HMGB1 expression.

In addition, this study also observed that RS postconditioning was able to decrease the level of MDA (a reactive oxygen species and an index for oxidative stress) and increase the activity of SOD (a key antioxidant enzyme). Previous studies indicated that oxidative stress may be involved in the release of HMGB1. Tang *et al*(19) showed that hydrogen peroxide, a reactive oxygen species, was able to stimulate macrophages and monocytes to actively release HMGB1. Tsung *et al*(20) further confirmed that the release of HMGB1 from cultured hepatocytes is an active process regulated by reactive oxygen species, indicating that the inhibition of reactive oxygen species may inhibit HMGB1 expression. These results suggest that postconditioning with RS may reduce HMGB1 expression, which may be associated with the inhibition of reactive oxygen species induced by myocardial I/R.

In this study, only the effect of RS postconditioning on myocardial I/R injury and HMGB1 expression was investigated. The precise mechanisms underlying our observations require future investigation.

The present study suggests that postconditioning with RS is able to reduce myocardial I/R injury, which may be associated with the inhibition of HMGB1 expression. These results show that RS may be useful for reducing myocardial I/R injury in clinical practice.
